# Respiratory Syncytial Virus and US Pediatric Intensive Care Utilization

**DOI:** 10.1001/jamanetworkopen.2024.40997

**Published:** 2024-10-25

**Authors:** Alice Shanklin, Taylor Olson, Anita K. Patel, Eduardo A. Trujillo Rivera, Murray M. Pollack

**Affiliations:** 1 Division of Pediatric Critical Care Medicine, Children’s National Hospital, Washington, DC; 2 Now with Division of Pediatric Critical Care Medicine, Cohen Children’s Medical Center, New York, New York; 3 Research Division of Biostatistics and Study Methodology, Children’s National Hospital, Washington, DC

## Abstract

**Importance:**

Bronchiolitis is the most common diagnosis necessitating respiratory support and pediatric intensive care, and respiratory syncytial virus (RSV) is the most common cause of bronchiolitis. In 2023, the monoclonal antibody nirsevimab and the maternal RSVpreF vaccine were implemented to prevent RSV in infants.

**Objective:**

To determine the potential association of novel RSV prevention strategies with pediatric intensive care unit (ICU) utilization.

**Design, Setting, and Participants:**

This retrospective cross-sectional study examined US pediatric ICU encounters with and without RSV from January 1, 2017, to June 1, 2023. Data were from Oracle Cerner RealWorld Data, a national electronic health record database. All children (aged >1 day and <18 years) admitted to an ICU during the study period were included in the analysis. Statistical analysis was performed from February to May 2024.

**Main Outcomes and Measures:**

The primary outcomes were ICU encounters with RSV and ICU encounters with RSV eligible for RSV prevention.

**Results:**

There were 119 782 ICU encounters from 53 hospitals; 65 757 encounters (54.9%) were male; median (IQR) age was 4.5 (1.1-12.5) years, median (IQR) ICU length of stay was 1.8 (1.0-3.9) days; 13 702 ICU encounters (11.4%) had RSV, of which 38.6% (5217) were eligible for prevention (4.4% of total ICU encounters). Encounters with RSV accounted for 21.1% (109 334) of ICU days, of which 43.8% (47 888) were eligible for prevention (9.2% of total ICU days). Encounters with RSV were associated with a greater use of positive pressure ventilation (4074 [29.7%] vs 18 821 [17.7%]; *P* < .001) and vasoactive medications (3057 [22.3%] vs 18 570 [17.5%]; *P* < .001) compared with those without RSV.

**Conclusions and Relevance:**

The findings of this retrospective cross-sectional study of RSV and US pediatric intensive care utilization suggest that if 65% to 85% of eligible children received RSV prevention, an estimated 2.1% to 2.8% reduction in pediatric ICU encounters and an estimated 4.5% to 5.9% reduction in pediatric ICU days could be achieved.

## Key Points

**Question:**

What proportion of pediatric intensive care unit (ICU) admissions are associated with respiratory syncytial virus (RSV) infections, and could nirsevimab and the maternal RSVpreF vaccine be associated with pediatric intensive care utilization?

**Findings:**

In this cross-sectional study of 119 782 pediatric ICU encounters, 11.4% had RSV, of which 38.6% were eligible for RSV prevention. If typical vaccine uptake were achieved, pediatric ICU encounters would be reduced by an estimated 2.1% to 2.8%, and intensive care unit days would be reduced by an estimated 4.5% to 5.9%.

**Meaning:**

These results suggest that nirsevimab and the maternal RSVpreF vaccine could be associated with a decrease in pediatric ICU utilization.

## Teaser

This cross-sectional study examines the potential association of novel respiratory syncytial virus (RSV) prevention strategies with pediatric intensive care unit utilization among children younger than 18 years of age.

## Introduction

Vaccines have had an important impact in preventing morbidity and mortality from serious infections, especially in children.^1,2^ Following successful vaccination programs, infectious diseases that accounted for major mortality and morbidity in the early 20th century declined more than 90% from their peak incidences in the US.^2^ This reduction in morbidity and mortality also led to substantial health care savings.^3^ In the 21st century, bronchiolitis is the most common pediatric diagnosis necessitating respiratory support and intensive care.^4^ Respiratory syncytial virus (RSV), the most common viral cause of bronchiolitis, accounts for an estimated 3.6 million hospital admissions annually for children younger than 5 years and contributes substantially to pediatric intensive care unit (ICU) utilization, morbidity, and mortality.^5,6^ RSV infection during infancy is also independently associated with childhood asthma and contributes to ICU utilization after the initial infection.^6^

Two RSV prevention strategies were implemented in the US in 2023. First, in July 2023, nirsevimab (Beyfortus [Sanofi]), an RSV monoclonal antibody with an extended half-life, was approved for infants under 8 months of age.^7,8^ Second, in August 2023, the recombinant, single-dose RSVpreF vaccine (Abrysvo [Pfizer]) was approved for pregnant people 32 to 36 weeks gestation to replace nirsevimab in full-term infants.^9^ Nirsevimab and the maternal RSVpreF vaccine were 83% and 67% effective at preventing hospitalization, respectively.^10,11^ This has also been observed by European countries that adopted nirsevimab early. Its efficacies at preventing ICU admission ranged from 70% to 86.9% in these settings.^12,13,14,15,16^ In the US, vaccine uptake is estimated to be between 65% and 85%.^17,18,19^ While prior studies established the effect of RSV infection on inpatient care needs in the US, the effect of RSV infection on pediatric ICU resource utilization is not known.^20,21^

This study is a national assessment of the monoclonal antibody nirsevimab and the maternal RSVpreF vaccine and their potential association with pediatric ICU resource utilization in the US.^13,14,22^ We conducted a retrospective analysis of a national multicenter database to determine the proportion of pediatric ICU encounters with RSV infection and the proportion of ICU encounters with RSV infection eligible for RSV prevention. We also explored the differences in clinical variables and markers of illness severity between these groups.

## Methods

### Data Source

This was a multicenter, retrospective cross-sectional study of Oracle’s Cerner RealWorld Data (CRWD), a deidentified national database of US hospitals.^23^ The database includes administrative and clinical data from the electronic health record (EHR), including diagnosis codes, medication administration, laboratory values, respiratory support, and hospital outcomes. Prior studies have used CRWD in pediatric and critical care settings.^24,25,26^ The Children’s National Hospital institutional review board approved the study with a waiver of informed consent because data were deidentified. This manuscript followed the Strengthening the Reporting of Observational Studies in Epidemiology (STROBE) reporting guideline.^27^

### Study Population, Variables, and Outcome Measures

Inclusion criteria were age greater than 1 day to younger than 18 years and admission to an ICU between January 1, 2017, and June 1, 2023. NICU encounters were excluded and planned surgical admissions were not excluded. Encounter variables collected or analyzed included admission date, age, weight, sex, insurance type, ICU and hospital length of stay, hospital type, and outcome (eTable 1 in Supplement 1). RSV infections were identified by RSV polymerase chain reaction or antigen test results or the presence of an RSV diagnosis code (*International Statistical Classification of Diseases, Tenth Revision, Clinical Modification [ICD-10-CM]* codes: B974, J20.5, J12.1, or J21.0).^28^

Illness severity was categorized as respiratory failure, positive pressure ventilation (PPV), vasoactive medication, extracorporeal membrane oxygenation (ECMO), and death. Respiratory failure was identified by *ICD-10-CM* code (eTable 2 in Supplement 1). PPV included invasive and noninvasive mechanical ventilation and was identified by procedure codes and/or a charted ventilator setting (eTables 3 and 4 in Supplement 1). Receipt of vasoactive medication was determined from the medication administration record (eTable 5 in Supplement 1). ECMO was determined from procedure codes (eTable 6 in Supplement 1).

Encounters were classified as eligible for RSV prevention if they met the Centers for Disease Control and Prevention Health Alert Network’s 2023 to 2024 recommendations for nirsevimab administration: the child was younger than 1 year on admission or younger than 2 years on admission with a condition that places them at high risk for severe disease.^9^ *ICD-10-CM* diagnosis codes were used to identify high-risk conditions (eTable 7 in Supplement 1).^9^

Encounters admitted from 2017 to 2019 were used to estimate the RSV peak and RSV season to minimize the disruption of seasonality trends by the COVID-19 pandemic. Encounters were defined as occurring in RSV peak and RSV season. The North American RSV season was defined as October through April, and the North American RSV peak was defined as December and January.^29,30^

The estimated outcome of the RSV prevention strategies was determined by their expected efficacy and uptake. A 75% combined efficacy was used to represent nirsevimab (83.2% efficacy) and RSVpreF vaccine (63.7% efficacy) at preventing hospital admissions.^10,11^ Patient-level utilization was estimated to be between 65% and 85% based on national surveys of parental vaccine hesitancy and COVID-19 vaccine uptake.^17,18^

### Statistical Analysis

Descriptive statistics (median [IQR] and number [percentage]) are provided for the variables of interest. Variables of interest were assessed in relation to encounter RSV infection status, and among encounters with RSV infection, variables were assessed in relation to RSV prevention eligibility status. Bivariate tests included Pearson χ^2^ test for categorical variables and the Wilcoxon rank sum test for continuous variables. Post hoc multiple comparisons were performed if the primary comparison was significant. All tests were 2-tailed, and a significance level of *P* ≤ .05 was used. All statistical analyses were conducted using JMP version 16.1 (JMP Statistical Discovery LLC) from February to May 2024.

## Results

### Cohort Description

There were 119 782 ICU encounters from 53 hospitals during the study period (Table 1). The median (IQR) age was 4.5 (1.1-12.5) years, median (IQR) weight was 17.0 (9.4-43.5) kg, and 65 757 patients (54.9%) were male. ICU length of stay was 1.8 days (1.0-3.9), and hospital length of stay was 3.7 days (2.0-7.3). Approximately half of encounters (51.4%) took place in a children’s hospital, and 48.6% had public insurance. Hospital type, size, and location are further detailed in eTable 8 and the eFigure in Supplement 1. Respiratory failure was present in 36.9% (44 200 of 119 782) of all ICU encounters, 19.1% (22 895 of 119 782) required positive pressure ventilation, 18.1% (21 627 of 119 782) received vasoactive medications, and 4.9% (5854 of 119 782) died. High-risk conditions were identified in 19.1% (22 862 of 119 782), and the most frequent high-risk condition was congenital heart disease (11.8% [14 178 of 119 782]). Age-specific data are listed in eTables 9, 10, and 11 in Supplement 1.

**Table 1.** Demographics and Outcomes for All Pediatric ICU Encounters Between January 1, 2017, and June 1, 2023

Variables	Encounters, No. (% total)			*P* value^a^
	Total (N = 119 782)	RSV (n = 13 702)	No RSV (n = 106 080)	
Sex				
Female	54025 (45.1)	5918 (43.2)	48 107 (45.4)	.26
Male	65 757 (54.9)	7784 (56.8)	57 973 (54.6)	
Age, median (IQR), y	4.5 (1.1-12.5)	1.8 (0.6-6.0)	5.1 (1.2-12.9)	<.001
Weight, median (IQR), kg	17.0 (9.4-43.5)	11.4 (7.5-21.2)	18.5 (9.9-45.9)	<.001
Primary insurance				
Public	58 197 (48.6)	7011 (51.2)	51 186 (48.3)	NA
Private	28 291 (23.6)	2761 (20.2)	25 530 (24.1)	<.001^b^
Other or unknown	33 294 (27.8)	3930 (28.7)	29 364 (27.7)	.28^b^
Hospital type^c^				
Children’s hospital	61 512 (51.4)	7118 (51.9)	58 314 (55.0)	.14
Nonchildren’s hospital	58 270 (48.6)	6584 (48.1)	51 686 (45.0)	
ICU length of stay				
ICU days per encounter, median (IQR)	1.8 (1.0-3.9)	2.8 (1.5-6.2)	1.7 (0.9-3.7)	<.001
Sum of ICU days (% row)	517 779 (100)	109 334 (21.1)	408 445 (78.9)	
Hospital length of stay				
Hospital days per encounter, median (IQR)	3.7 (2.0-7.3)	5.5 (3.0-11.9)	3.5 (2.0-7.0)	<.001
Sum of hospital days (% row)	930 092 (100)	189 408 (20.4)	740 684 (79.6)	
Illness severity				
Respiratory failure	44 200 (36.9)	9723 (70.9)	34 477 (32.5)	<.001
Positive pressure ventilation^d^	22 895 (19.1)	4074 (29.7)	18 821 (17.7)	<.001
Vasoactive medication	21 627 (18.1)	3057 (22.3)	18 570 (17.5)	<.001
ECMO	387 (0.32)	124 (0.9)	263 (0.3)	<.001
Death	5854 (4.9)	735 (5.3)	5119 (4.8)	.006
High-risk condition	22 862 (19.1)	2561 (18.7)	20 301 (19.1)	.21
High-risk condition^e^				
Chronic respiratory failure	3436 (2.9)	388 (2.8)	3048 (2.9)	.78
Perinatal respiratory disease	140 (0.1)	10 (0.1)	130 (0.1)	.11
Congenital heart disease	14 178 (11.8)	1336 (9.7)	12 842 (12.1)	<.001
Prematurity	3418 (2.8)	493 (3.6)	2925 (2.7)	<.001
Immunodeficiency	2748 (2.3)	561 (4.1)	2187 (2.1)	<.001
Muscular dystrophy	291 (0.2)	28 (0.2)	263 (2.5)	.33
Cystic fibrosis	256 (0.2)	29 (0.2)	227 (2.1)	.96
Neuromuscular scoliosis	1522 (1.3)	113 (0.8)	1409 (1.3)	<.001

Abbreviations: ECMO, extracorporeal membrane oxygenation; ICU, intensive care unit; NA, not applicable; RSV, respiratory syncytial virus.

^a^ RSV and non-RSV encounter characteristics were compared using 2-tailed Pearson χ^2^ tests for categorical variables and 2-tailed Wilcoxon rank-sum tests for continuous variables.

^b^ Primary, public, and unknown insurance types were compared with post hoc analysis. Public insurance was the reference group.

^c^ The percentage of total encounters in the children’s and nonchildren’s hospital groups is shown.

^d^ Positive pressure ventilation includes both invasive and noninvasive ventilation.

^e^ High-risk conditions were identified by *International Statistical Classification of Diseases, Tenth Revision, Clinical Modification* codes selected based on the Centers for Disease Control and Prevention recommendations for nirsevimab administration; see Supplement 1 for classification.^9^

### Prevalence and Resource Utilization Associated With RSV Infection

**Figure 1.**
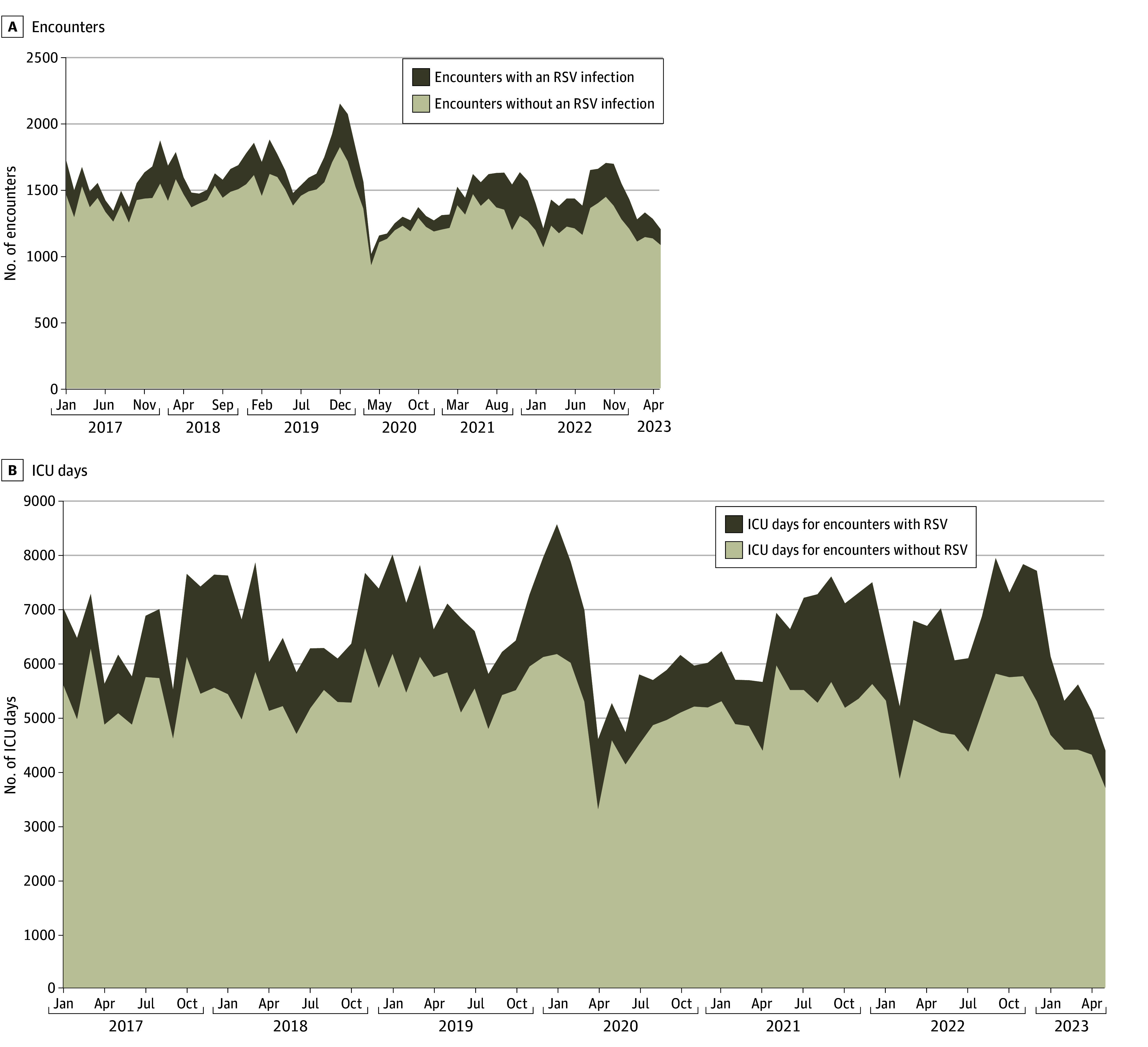
Encounters and Intensive Care Unit (ICU) Days by Admission Month RSV indicates respiratory syncytial virus.

RSV infection was identified in 13 702 ICU encounters (11.4%) (Table 1, Figure 1). Encounters with RSV infections were distributed similarly between children’s hospitals and nonchildren’s hospitals (7118 [51.9%] vs 58 314 [48.1%]; *P* = .14). Encounters with RSV infections had a lower median (IQR) age compared with encounters without RSV (1.8 [0.6-6.0] years vs 5.1 [1.2-12.9] years; *P* < .001) and were more likely to have public insurance (7011 [51.2%] vs 51 186 [48.3%]; *P* < .001). Encounters with RSV infections accounted for 21.1% of the total ICU days and 20.4% of the total hospital days (Table 1). During the North American RSV peak (December and January), 14.7% of all encounters had an RSV infection, accounting for 21.1% of all ICU days and 22.9% of all hospital days (Table 2). During this time 22.2% of all encounters with patients aged 0 to 2 years had an RSV infection, accounting for 30.1% of ICU days and 30.0% of hospital days of this age group (eTable 9 in Supplement 1).

**Table 2.** Seasonality of All Pediatric ICU Encounters Between January 1, 2017, and December 31, 2019

Variables	Encounters		
	Total	RSV	No RSV
**All ICU encounters 2017-2019^a^**			
Encounters, No. (%)	58 902 (100)	5945 (10.1)	52 957 (89.9)
ICU days, sum (%)	244 386 (100)	47 818 (19.6)	196 567 (80.4)
Hospital days, sum (%)	441 870 (100)	81 245 (18.3)	360 625 (81.6)
**RSV season^b^**			
Encounters, No. (%)	36 252 (100)	4432 (12.2)	31 820 (87.8)
ICU days, sum (%)	149 767 (100)	31 625 (21.1)	118 142 (78.8)
Hospital days, sum (%)	270 525 (100)	54 680 (20.2)	215 845 (79.8)
**RSV peak^b^**			
Encounters, No. (%)	11 029 (100)	1618 (14.7)	9411 (85.3)
ICU days, sum (%)	45 550 (100)	11 197 (24.6)	34 353 (75.4)
Hospital days, sum (%)	82 707 (100)	18 900 (22.9)	63 807 (77.1)

Abbreviations: ICU, intensive care unit; RSV, respiratory syncytial virus.

^a^ Encounters admitted from 2017-2019 were used to represent the RSV season and the RSV peak to minimize alteration by the COVID-19 pandemic.^45^

^b^ North American RSV season is defined as October-April. The peak is defined as December to January.^31^

RSV infection was associated with greater illness severity including longer median (IQR) ICU length of stay (2.8 [1.5-6.2] vs 1.7 [0.9-3.7] days; *P* < .001), median (IQR) hospital length of stay (5.5 [3.0-11.9] vs 3.5 [2.0-7.0] days; *P* < .001); higher proportion of respiratory failure (9723 [70.9%] vs 34 477 [30.5%]; *P* < .001), positive pressure ventilation (4074 [29.7%] vs 18 821 [17.7%]; *P* < .001), vasoactive medication (3057 [22.3%] vs 18 570 [17.5%]; *P* < .001); ECMO (124 [0.9%] vs 263 [0.3%]; *P* < .001) and death (735 [5.3%] vs 5119 [4.8%]; *P* = .006) compared with encounters without RSV (Table 1).

### Potential Association With Pediatric ICU Resource Utilization

Of the cohort, 13 702 ICU encounters (11.4%) had RSV infection, of which 5217 (38.6%) were eligible for RSV prevention. Encounters with RSV infections accounted for 21.1% of ICU days, of which 43.8% were eligible for RSV prevention. Therefore, 4.4% of ICU encounters and 9.2% of ICU days represent encounters that have RSV and are eligible for RSV prevention. Encounters with patients aged 0 to 2 years with RSV infection accounted for 10.9% of ICU days and 72.3% were eligible for RSV prevention (eTables 10 and 11 in Supplement 1). Encounters with RSV infections who were not eligible for RSV prevention were associated with more severe illness than those eligible for prevention, with more PPV (2573 [30.3%] vs 1501 [28.8%]; *P* < .001), vasoactive medication use (2060 [24.3%] vs 997 [19.1%]; *P* < .001), and deaths (545 [6.4%] vs 190 [4.8%]; *P* < .001) (Table 3). The results for PPV were consistent in age groups 0 to 2 years, 2 to 5 years, and greater than 5 years (eTable 10 in Supplement 1). Mortality was different between age groups. RSV was associated with decreased mortality in the age group of 0 to 2 years (251 [3.5%] vs 1461 [4.2%]; *P* = .005), not associated with mortality in the aged group of 2 to 5 years (149 [5.8%] vs 917 [5.2%]; *P* = .21), and associated with increased mortality in the age group of 5 years and older (335 [8.7%] vs 2741 [5.1%]; *P* < .001) (eTable 10 in Supplement 1).

**Table 3.** Eligibility for RSV Prevention Among Pediatric ICU Encounters With an RSV Infection Between January 1, 2017, and June 1, 2023

Variables	RSV infection present, No. (%)		*P* value^b^
	Eligible for RSV prevention^a^	Not eligible for RSV prevention^a^	
Total encounters^c^	5217 (38.1)	8485 (61.9)	NA
Hospital type			
Children’s hospital	2563 (35.7)	4555 (63.4)	<.001
Nonchildren’s hospital	2654 (40.3)	3930 (59.6)	
ICU days			
Median (IQR)	3.2 (1.8-6.8)	2.6 (1.4-5.8)	<.001
Sum (% total ICU days)	47 888 (43.8)	61 446 (56.2)	
Hospital days			
Median (IQR)	5.9 (3.6-11.9)	5.1 (2.9-11.9)	<.001
Sum (% total hospital days)	75 030 (39.6)	114 378 (60.4)	
Illness severity			
Respiratory failure	4023 (77.1)	5700 (67.2)	<.001
Positive pressure ventilation^d^	1501 (28.8)	2573 (30.3)	.05
Vasoactive medication	997 (19.1)	2060 (24.3)	<.001
ECMO	54 (0.1)	70 (0.1)	.21
Death	190 (3.6)	545 (6.4)	<.001

Abbreviations: ECMO, extracorporeal membrane oxygenation; ICU, intensive care unit; NA, not applicable; RSV, respiratory syncytial virus.

^a^ Encounters with patients under 1 year of age on admission and encounters with patients under 2 years of age on admission with a high-risk condition were considered eligible for RSV prevention in accordance with the Centers for Disease Control and Prevention (CDC) guidelines.^9^ High-risk conditions were identified by *International Statistical Classification of Diseases, Tenth Revision, Clinical Modification* codes based on the CDC’s recommendations for nirsevimab administration; see eAppendix in Supplement 1 for classification.

^b^ Encounters with RSV eligible for prevention and encounters with RSV not eligible for prevention were compared using 2-tailed Pearson tests for categorical variables and Wilcoxon rank-sum tests for continuous variables.

^c^ Percentage of total encounters with an RSV infection.

^d^ Positive pressure ventilation includes both invasive and noninvasive mechanical ventilation.

**Figure 2.**
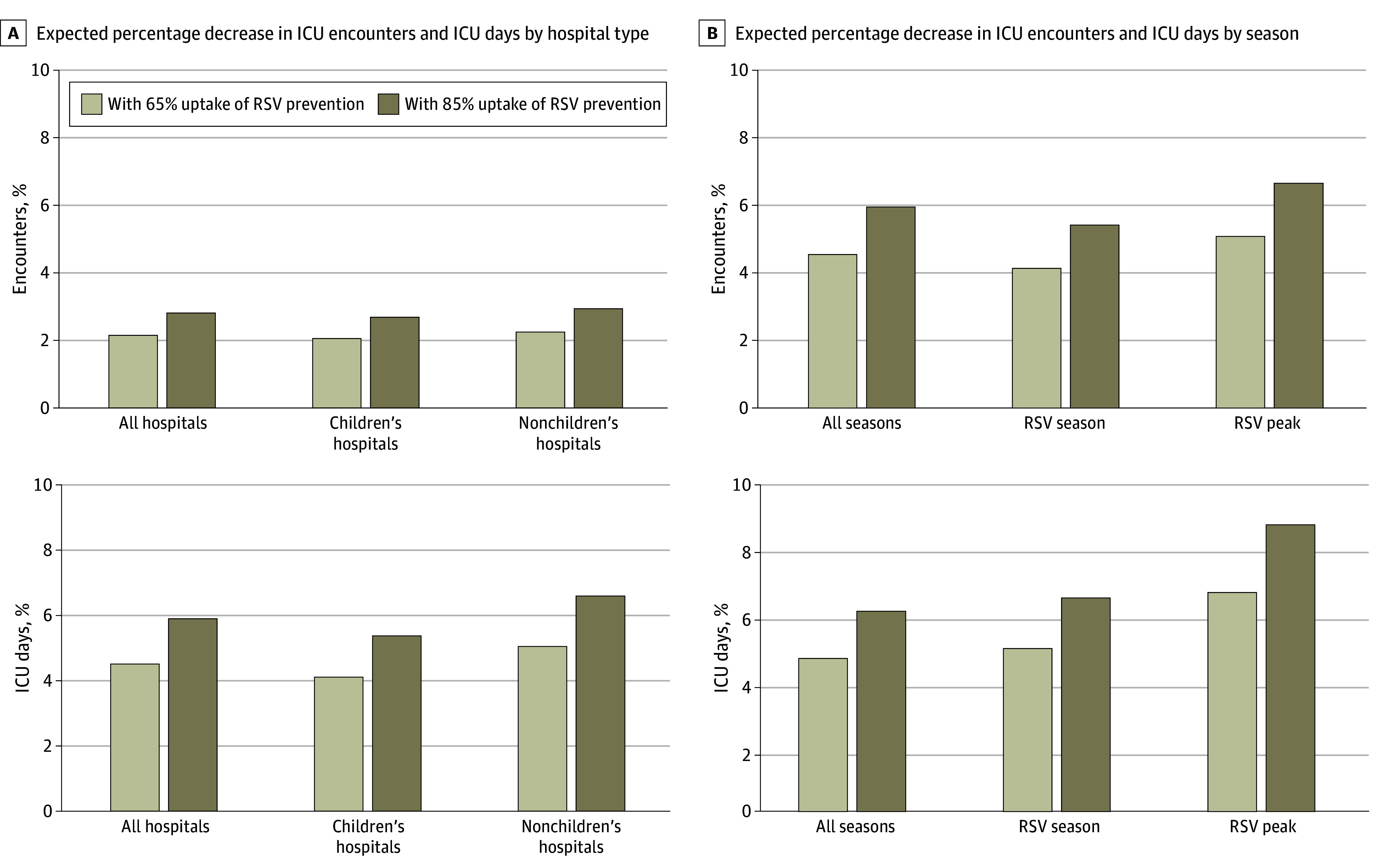
Estimated Percentage Decrease in Intensive Care Unit (ICU) Encounters and ICU Days A, Estimated percent decrease in ICU encounters and ICU days with 65% and 85% uptake of the respiratory syncytial virus (RSV) prevention strategies. The estimated percentage decrease in total ICU encounters and total ICU days is illustrated by hospital type. B, The estimated percentage decrease in total ICU encounters and total ICU days by season. The North American RSV season is defined as October-April, and the peak is defined as December to January. Encounters admitted from 2017 to 2019 were used to estimate the RSV season and the RSV peak to minimize disruption by the COVID-19 pandemic.

With a 75% efficacy at preventing hospital admission and 65% to 85% vaccine uptake, ICU encounters would be associated with an estimated 2.1% to 2.8% reduction and ICU days would be associated with an estimated 4.5% to 5.9% reduction (Figure 2). During the North American RSV peak, ICU encounters would be associated with an estimated 3.6% to 4.7% reduction and ICU days would be associated with an estimated 6.7% to 8.8% reduction. The estimated percentage decrease in ICU encounters by hospital type and season is shown in Figure 2.

## Discussion

This study estimates the association of the new RSV preventive strategies with ICU utilization for children in the US. We examined 119 782 ICU encounters from the national multicenter CRWD database and identified that 11.4% had RSV infection, accounting for 21.1% of the total ICU days. RSV infection was associated with markers of greater severity of illness, including ICU length of stay, positive pressure ventilation, and death. Using 75% combined efficacy for reducing hospital admission and 65% to 85% uptake, we anticipate a 2.1% to 2.8% reduction in pediatric ICU encounters and a 4.5% to 5.9% reduction in ICU days.^10,11,12,17,18,19,31^

The novel preventive strategies for RSV follow a historical precedent of widespread vaccination. In the last century, the pertussis vaccine reduced the incidence of pertussis by over 90%,^32^ the Hemophilus influenzae type b (Hib) vaccine reduced the incidence of Hib meningitis in children under 5 years by over 90%,^33,34^ the pneumococcal vaccine reduced the incidence of invasive pneumococcal disease in US children by over 90%,^34,35^ the meningococcal vaccine reduced the incidence of meningococcal disease in United Kingdom adolescents by over 97%,^35^ and the oral rotavirus vaccine decreased hospitalization for diarrheal illnesses in eligible infants by over 50%.^36^ While it is too early to compare the RSV infection prevention strategies to these successes, they could emulate the effect of prior vaccination efforts.

The novel RSV prevention strategies could alter the recent trend of pediatric ICU expansion. Inpatient pediatric care has shifted from acute care beds to ICU beds over the past century, which has accelerated in recent years.^37^ Despite an overall decrease in inpatient pediatric care, pediatric ICU beds have increased by 46% in the past decade, increasing by 200 beds per year after 2016.^31,37^ Novel RSV prevention strategies could alter the demand for pediatric ICU beds.^30^ We conservatively estimated a 2.1% to 2.8% reduction in ICU encounters and a 4.5% to 5.9% reduction in ICU days, with a 6.7% to 8.8% reduction in ICU days at the RSV seasonal peak.^10,11,17,18^ Using a mean cost of $22 043 for each PICU encounter and 85% uptake, nirsevimab would have saved $14 772 340 per year on ICU care in our cohort.^38^

Vaccine uptake in the US is limited by access and acceptance. In the 2023 to 2024 season, pediatric clinics with higher proportions of privately insured patients and lower proportions of patients in lower-income zip codes were more likely to have access to nirsevimab.^39^ Acceptance of the therapy was also an issue. Only 47% of eligible patients at clinics with access to the drug received it, and patients from lower-income zip codes were less likely to do so.^39^ This experience indicates the potential for a substantial expansion of nirsevimab’s impact if these issues are addressed. If 100% of eligible infants receive nirsevimab, as they have in some parts of Spain, a 3.2% reduction in ICU encounters and a 6.9% reduction in ICU days may be possible.^12,31^

The novel RSV prevention strategies may also reduce pediatric ICU acuity. RSV infection was significantly associated with respiratory failure, positive pressure ventilation, and ECMO use in children aged 0 to 2 years, 2 to 5 years and greater than 5 years on admission (eTable 10 in Supplement 1). Additionally, 61.9% of encounters with RSV infection were not eligible for RSV prevention, and these encounters demonstrated greater illness severity than those eligible, with more positive pressure ventilation, vasoactive medication use, and death. Therefore, expanded eligibility for RSV prevention can potentially reduce ICU care utilization, morbidity, and mortality even further.

### Limitations

This study had limitations. The efficacies of nirsevemab and the maternal RSV preF vaccine used in this analysis were based on early studies, which is a key limitation of this manuscript. When updated uptake and efficacy data are published, a more robust estimation will be possible.^10,11^ This manuscript had several other limitations. It was not possible to determine whether the RSV infection was the primary reason for admission vs an incidental finding. This is particularly important in patients with chronic critical illness, as they are often admitted to the ICU for a longer period and, therefore, have more opportunities to test positive for RSV.^40,41^ RSV infections in encounters with laboratory testing before hospitalization may have been missed, resulting in an underestimation of the impact of these preventive strategies. We did not account for RSV infections in neonatal ICUs, although there is a risk of nosocomial RSV infection within this population.^42^ Additionally, we did not account for decreased community spread, which could decrease the care needs of patients with RSV infection who are not eligible for prevention.^43^

## Conclusions

In this retrospective cross-sectional study of RSV and US pediatric intensive care utilization, we estimated a 2.1% to 2.8% reduction in ICU encounters and a 4.5% to 5.9% reduction in ICU days if a 65% to 85% uptake of the novel RSV prevention strategies is achieved for eligible children, with a 3.6% to 4.7% reduction during the North American RSV Peak in December and January. Children with RSV infection who require ICU admission have a 29.7% chance of requiring positive pressure ventilation, a 22.3% chance of requiring vasoactive medications, and a 5.3% mortality. The novel RSV prevention strategies may reduce ICU morbidity and mortality for children. The results suggest that efforts to increase availability and acceptance of these therapies are warranted. Further expansion of the population eligible for RSV prevention could have a substantial impact.

## Notes

Supplement 1. **eTable 1.** Insurance Designations and Their Classification as Public, Private, or ‘Not Available’ **eTable 2.** ICD-10 Diagnosis Codes Used to Identify Acute or Unspecified Respiratory Failure **eTable 3.** ICD-10 Procedure Codes Used to Identify Positive Pressure or Mechanical Ventilation **eTable 4.** ICD-10 Clinical Events Used to Identify Noninvasive and Invasive Positive Pressure Ventilation **eTable 5.** Medication Administration Drug IDs Used to Identify the Administration of a Vasoactive Medication **eTable 6.** ICD-10 Procedure Codes Used to Identify ECMO **eTable 7.** International Classification of Diseases, Tenth Revision (ICD-10) Codes Used to Identify High-Risk Conditions, Defined as Medical Conditions That Make Patients Eligible For Respiratory Syncytial Virus (RSV) Prevention in Their 2nd RSV Season per the CDC Recommendations **eTable 8.** Hospital Type, Size, and Location of All Pediatric ICU Encounters Between January 1st, 2017, and December 31st, 2019, Stratified by RSV Infection **eTable 9.** Seasonality of All Pediatric ICU Encounters Between January 1st, 2017, and December 31st, 2019, Stratified by Age **eTable 10.** Demographics and Outcomes for All Pediatric ICU Encounters Between January 1st, 2017, and June 1st, 2023, Stratified by Age **eTable 11.** Demographics and Outcomes for All Pediatric ICU Encounters With RSV Between January 1st, 2017, and June 1st, 2023, Stratified by Positive Pressure Ventilation and Age **eFigure.** Number of Encounters in Each United States Zip Code Region
eReferences.

Supplement 2.
Data Sharing Statement
