# Error in Introduction

**DOI:** 10.1001/jamanetworkopen.2025.4977

**Published:** 2025-03-03

In the Original Investigation titled “Respiratory Syncytial Virus and US Pediatric Intensive Care Utilization,”^1^ published October 25, 2024, the maternal RSVpreF vaccine was referred to by the wrong brand name; it should have been “Abrysvo.” This article has been corrected.^1^
