# Supplementary material for: Respiratory Syncytial Virus and US Pediatric Intensive Care Utilization
Source: JAMA Netw Open. 2024 Oct 25;7(10):e2440997. doi: 10.1001/jamanetworkopen.2024.40997 (PMC11581606; doi:10.1001/jamanetworkopen.2024.40997)
Supplement: Supplement 1. — eTable 1. Insurance Designations and Their Classification as Public, Private, or ‘Not Available’ eTable 2. ICD-10 Diagnosis Codes Used to Identify Acute or Unspecified Respiratory Failure eTable 3. ICD-10 Procedure Codes Used to Identify Positive Pressure or Mechanical Ventilation eTable 4. ICD-10 Clinical Events Used to Identify Noninvasive and Invasive Positive Pressure Ventilation eTable 5. Medication Administration Drug IDs Used to Identify the Administration of a Vasoactive Medication eTable 6. ICD-10 Procedure Codes Used to Identify ECMO eTable 7. International Classification of Diseases, Tenth Revision (ICD-10) Codes Used to Identify High-Risk Conditions, Defined as Medical Conditions That Make Patients Eligible For Respiratory Syncytial Virus (RSV) Prevention in Their 2nd RSV Season per the CDC Recommendations eTable 8. Hospital Type, Size, and Location of All Pediatric ICU Encounters Between January 1st, 2017, and December 31st, 2019, Stratified by RSV Infection eTable 9. Seasonality of All Pediatric ICU Encounters Between January 1st, 2017, and December 31st, 2019, Stratified by Age eTable 10. Demographics and Outcomes for All Pediatric ICU Encounters Between January 1st, 2017, and June 1st, 2023, Stratified by Age eTable 11. Demographics and Outcomes for All Pediatric ICU Encounters With RSV Between January 1st, 2017, and June 1st, 2023, Stratified by Positive Pressure Ventilation and Age eFigure. Number of Encounters in Each United States Zip Code Region eReferences. [file jamanetwopen-e2440997-s001.pdf]

Supplemental Online Content

Shanklin A, Olson T, Patel AK, Trujillo Rivera EA, Pollack MM. Respiratory syncytial virus and United States pediatric intensive care utilization. *JAMA Netw Open.* 2024;7(10):e2440997. doi:10.1001/jamanetworkopen.2024.40997

eTable 1. Insurance Designations and Their Classification as Public, Private, or ‘Not Available’

eTable 2. ICD-10 Diagnosis Codes Used to Identify Acute or Unspecified Respiratory Failure

eTable 3. ICD-10 Procedure Codes Used to Identify Positive Pressure or Mechanical Ventilation

eTable 4. ICD-10 Clinical Events Used to Identify Noninvasive and Invasive Positive Pressure Ventilation

eTable 5. Medication Administration Drug IDs Used to Identify the Administration of a Vasoactive Medication

eTable 6. ICD-10 Procedure Codes Used to Identify ECMO

eTable 7. International Classification of Diseases, Tenth Revision (ICD-10) Codes Used To Identify High-Risk Conditions, Defined as Medical Conditions That Make Patients Eligible For Respiratory Syncytial Virus (RSV) Prevention in Their 2nd RSV Season per the CDC Recommendations

eTable 8. Hospital Type, Size, and Location of All Pediatric ICU Encounters Between January 1st, 2017, and December 31st, 2019, Stratified by RSV Infection

eTable 9. Seasonality of All Pediatric ICU Encounters Between January 1st, 2017, and December 31st, 2019, Stratified by Age

eTable 10. Demographics and Outcomes for All Pediatric ICU Encounters Between January 1st, 2017, and June 1st, 2023, Stratified by Age

eTable 11. Demographics and Outcomes for All Pediatric ICU Encounters With RSV Between January 1st, 2017, and June 1st, 2023, Stratified by Positive Pressure Ventilation and Age

eFigure. Number of Encounters in Each United States Zip Code Region

eReferences

This supplemental material has been provided by the authors to give readers additional information about their work.

© 2024 Shanklin A et al. *JAMA Network Open.*

Supplemental Methods

The primary insurer for each encounter was classified as 'private,' 'public,' or 'other/unknown.' Appendix Table 1 shows insurance designations and the number of patients who meet each description.

The illness severity of each encounter was determined by ICD-10 diagnosis codes, ICD-10 procedure codes, 'clinical events' or clinical documentation, and the medication administration record. Appendix Tables 2-6 list the specific CRWD codes and designations used for this classification. Respiratory failure was identified by the presence of an ICD-10 diagnosis code (Appendix Table 2). Noninvasive and/or invasive positive pressure ventilation were identified using procedure codes (Appendix Table 3) and/or clinical events (Appendix Table 4). Vasoactive medication administration was identified by the presence of a vasoactive medication in the medication administration record (Appendix Table 5). Extracorporeal membrane oxygenation (ECMO) was identified using ICD-10 procedure codes (Appendix Table 6).

All encounters were designated as 'eligible for RSV prevention' or 'not eligible for RSV prevention,' according to the CDC's 2023 recommendations for nirsevimab and the maternal RSVpreF vaccine.[2–6] The CDC's 2023 recommendations indicate that all infants should receive RSV prevention for their first RSV season and infants with a designated high-risk condition should receive RSV prevention for their second RSV season.[2] All encounters admitted under one year of age were considered eligible for RSV prevention. Encounters with a designated high-risk condition admitted under two years of age were also considered eligible for RSV prevention. All ICD-10 diagnosis codes representing these high-risk conditions were identified and classified. The complete list of ICD-10 codes and classifications is shown in Appendix Table 1.

eTable 1: Insurance designations, insurance classifications, and the number of encounters with each designation in the database.

DATABASE Designation	Insurance Type	N
MEDICAID	Public	29490
Medicaid (Managed Care)	Public	10453
Medicaid HMO	Public	9242
OTHER GOVERNMENT (Federal/State/Local) (excluding Department of Corrections)	Public	4613
TRICARE (CHAMPUS)	Public	1979
Medicaid - Out of State	Public	1196
Medicaid Managed Care Other	Public	652
MEDICARE	Public	510
Pending Medicaid coverage	Public	36
Charity	Public	14
Department of Veterans Affairs	Public	7
DEPARTMENTS OF CORRECTIONS	Public	3
Medicare (Managed Care)	Public	2
PRIVATE HEALTH INSURANCE	Private	16515
BLUE CROSS/BLUE SHIELD	Private	7743
Managed Care (Private)	Private	738
BC Managed Care	Private	666
Foreign National	Private	482

Other (Non-government)	Private	329
Auto Insurance (no fault) 2217 BC Indemnity	Private	68
Worker's Compensation	Private	39
BC Managed Care - HMO	Private	16
Research/Donor	Private	15
NA	Other or Unknown	20297
HMO	Other or Unknown	6471
PPO	Other or Unknown	3285
Self-pay	Other or Unknown	2472
POS	Other or Unknown	95
Exclusive Provider Organization	Other or Unknown	67
Managed Care, Other (non HMO)	Other or Unknown	58
MISCELLANEOUS/OTHER	Other or Unknown	12

eTable 2: ICD-10 diagnosis codes used to identify acute or unspecified respiratory failure.

Code ID	Primary Display	ID Description
J96.01	acute respiratory failure with hypoxia	IC10
J96.00	acute respiratory failure, unspecified whether with hypoxia or hypercapnia	IC10
J96.21	acute and chronic respiratory failure with hypoxia	IC10
J96.90	respiratory failure, unspecified, unspecified whether with hypoxia or hypercapnia	IC10
J96.02	acute respiratory failure with hypercapnia	IC10
J96.20	acute and chronic respiratory failure, unspecified whether with hypoxia or hypercapnia	IC10
J95.821	acute postprocedural respiratory failure	IC10
J96.91	respiratory failure, unspecified with hypoxia	IC10
J96.22	acute and chronic respiratory failure with hypercapnia	IC10
J96.0	acute respiratory failure	IC10
J96.92	respiratory failure, unspecified with hypercapnia	IC10

eTable 3: ICD-10 Procedure codes used to identify positive pressure or mechanical ventilation

Code ID	Primary Display
5A1955Z	respiratory ventilation, greater than 96 consecutive hours
5A1945Z	respiratory ventilation, 24-96 consecutive hours
5A1935Z	respiratory ventilation, less than 24 consecutive hours
5A09357	assistance with respiratory ventilation, less than 24 consecutive hours, continuous positive airway pressure
5A09457	assistance with respiratory ventilation, 24-96 consecutive hours, continuous positive airway pressure
5A09557	assistance with respiratory ventilation, greater than 96 consecutive hours, continuous positive airway pressure
5A0945Z	assistance with respiratory ventilation, 24-96 consecutive hours

© 2024 Shanklin A et al. *JAMA Network Open.*

5A0935Z	assistance with respiratory ventilation, less than 24 consecutive hours
5A0955Z	assistance with respiratory ventilation, greater than 96 consecutive hours
5A09358	assistance with respiratory ventilation, less than 24 consecutive hours, intermittent positive airway pressure
5A09458	assistance with respiratory ventilation, 24-96 consecutive hours, intermittent positive airway pressure
5A09558	assistance with respiratory ventilation, greater than 96 consecutive hours, intermittent positive airway pressure
94002	ventilation assist and management, initiation of pressure or volume preset ventilators for assisted or controlled breathing; hospital inpatient/observation, initial day
94003	ventilation assist and management, initiation of pressure or volume preset ventilators for assisted or controlled breathing; hospital inpatient/observation, each subsequent day

eTable 4: ICD-10 Clinical events used to identify noninvasive and invasive positive pressure ventilation.

ID	Event Name	Event Class
20077-4	positive end expiratory pressure setting ventilator	Other
33446-6	airway temperature setting ventilator	Other
19840-8	breath rate spontaneous and mechanical --on ventilator	Other
2.51E+08	end tidal carbon dioxide concentration	Other
20079-0	pressure support setting ventilator	Other
19834-1	breath rate setting ventilator	Other
19839-0	breath rate spontaneous --on ventilator	Other
20116-0	tidal volume.spontaneous --on ventilator	Other
20112-9	tidal volume setting ventilator	Other
13621006	tidal volume	Other
38199-6	intrinsic peep respiratory system	Other
19994-3	oxygen/total gas setting volume fraction ventilator	Other
58959-8	bipap and/or cpap setting ventilator	Other
20058-4	airway pressure ventilator --at peak inspiratory flow setting	Other
76007-4	tidal volume expired respiratory system airway --on ventilator	Other
33438-3	breath rate mechanical --on ventilator	Other
19976-0	maximum pressure respiratory system airway opening --during inspiration on ventilator	Other
76530-5	mean pressure respiratory system airway --on ventilator	Other
60794-5	inspiratory gas flow respiratory system airway --on ventilator	Other
20075-8	peep respiratory system	Other
47545007	continuous positive airway pressure ventilation treatment	Other
20057-6	airway pressure ventilator --at peak inspiratory flow maximum setting	Other
20124-4	ventilation mode ventilator	Other

eTable 5: Medication administration drug IDs used to identify the administration of a vasoactive medication.

Coding System	Drug Code ID	Med Name	Route
314	d00704	phenylephrine	Intravenous
314	d00699	EPINEPHrine	Intravenous

© 2024 Shanklin A et al. *JAMA Network Open.*

314	d00302	milrinone	Intravenous
314	d00216	DOPamine	Intravenous
312	24063	EPINEPHrine 0.1 mg/mL injectable solution	Intravenous
312	12424	EPINEPHrine	Intravenous
312	198542	EPINEPHrine 10 mcg/mL-NaCl 0.9% intravenous solution	Intravenous
314	d00323	norepinephrine	Intravenous
314	d00398	vasopressin	Intravenous
312	23409	EPINEPHrine 1 mg/mL injectable solution	Intravenous
312	12216	milrinone	Intravenous
312	23078	milrinone 1 mg/mL intravenous solution	Intravenous
312	12429	phenylephrine	Intravenous
314	d00215	DOBUTamine	Intravenous
312	24422	phenylephrine 10 mg/mL injectable solution	Intravenous
312	12146	DOPamine	Intravenous
312	25671	DOPamine 1.6 mg/mL-D5% intravenous solution	Intravenous
312	12145	DOBUTamine	Intravenous
312	12296	vasopressin	Intravenous
312	25672	DOPamine 3.2 mg/mL-D5% intravenous solution	Intravenous
312	25698	milrinone 200 mcg/mL-D5% intravenous solution	Intravenous
312	24736	norepinephrine 1 mg/mL intravenous solution	Intravenous
314	d00704	phenylephrine	Intravenous central route
312	12232	norepinephrine	Intravenous
312	23510	vasopressin 20 units/mL injectable solution	Intravenous
312	194335	phenylephrine 100 mcg/mL-NaCl 0.9% intravenous solution	Intravenous
312	26302	DOBUTamine 1 mg/mL-D5% intravenous solution	Intravenous
312	194333	phenylephrine 20 mg/250 mL-NaCl 0.9% intravenous solution	Intravenous
314	d00302	milrinone	Intravenous central route
312	147616	phenylephrine 1 mg/10 mL-NaCl 0.9% intravenous solution	Intravenous
312	25345	DOPamine 800 mcg/mL-D5% intravenous solution	Intravenous
312	26303	DOBUTamine 2 mg/mL-D5% intravenous solution	Intravenous
314	d00704	phenylephrine	Intravenous central
312	26304	DOBUTamine 4 mg/mL-D5% intravenous solution	Intravenous
314	d00302	milrinone	Intravenous central

eTable 6: ICD-10 procedure codes used to identify ECMO.

Code ID	Tag	Primary Display
5A1522G	ECMO	extracorporeal oxygenation, membrane, peripheral veno-arterial
5A1522H	ECMO	extracorporeal oxygenation, membrane, peripheral veno-venous
5A15223	ECMO	extracorporeal membrane oxygenation, continuous
5A1522F	ECMO	extracorporeal oxygenation, membrane, central

© 2024 Shanklin A et al. *JAMA Network Open.*

33953	ECMO	extracorporeal membrane oxygenation (ecmo)/extracorporeal life support (ecls) provided by physician; insertion of peripheral (arterial and/or venous) cannula(e), open, birth through 5 years of age
33947	ECMO	extracorporeal membrane oxygenation (ecmo)/extracorporeal life support (ecls) provided by physician; initiation, veno-arterial
33969	ECMO	extracorporeal membrane oxygenation (ecmo)/extracorporeal life support (ecls) provided by physician; removal of peripheral (arterial and/or venous) cannula(e), open, birth through 5 years of age
33955	ECMO	extracorporeal membrane oxygenation (ecmo)/extracorporeal life support (ecls) provided by physician; insertion of central cannula(e) by sternotomy or thoracotomy, birth through 5 years of age
33985	ECMO	extracorporeal membrane oxygenation (ecmo)/extracorporeal life support (ecls) provided by physician; removal of central cannula(e) by sternotomy or thoracotomy, birth through 5 years of age
33954	ECMO	extracorporeal membrane oxygenation (ecmo)/extracorporeal life support (ecls) provided by physician; insertion of peripheral (arterial and/or venous) cannula(e), open, 6 years and older
2.33E+08	ECMO	cardiac support using extracorporeal membrane oxygenation circuitry
33946	ECMO	extracorporeal membrane oxygenation (ecmo)/extracorporeal life support (ecls) provided by physician; initiation, veno-venous
33984	ECMO	extracorporeal membrane oxygenation (ecmo)/extracorporeal life support (ecls) provided by physician; removal of peripheral (arterial and/or venous) cannula(e), open, 6 years and older
33949	ECMO	extracorporeal membrane oxygenation (ecmo)/extracorporeal life support (ecls) provided by physician; daily management, each day, veno-arterial
2.34E+08	ECMO	extracorporeal membrane oxygenation
33952	ECMO	extracorporeal membrane oxygenation (ecmo)/extracorporeal life support (ecls) provided by physician; insertion of peripheral (arterial and/or venous) cannula(e), percutaneous, 6 years and older (includes fluoroscopic guidance, when performed)
33959	ECMO	extracorporeal membrane oxygenation (ecmo)/extracorporeal life support (ecls) provided by physician; reposition peripheral (arterial and/or venous) cannula(e), open, birth through 5 years of age (includes fluoroscopic guidance, when performed)
33956	ECMO	extracorporeal membrane oxygenation (ecmo)/extracorporeal life support (ecls) provided by physician; insertion of central cannula(e) by sternotomy or thoracotomy, 6 years and older
33966	ECMO	extracorporeal membrane oxygenation (ecmo)/extracorporeal life support (ecls) provided by physician; removal of peripheral (arterial and/or venous) cannula(e), percutaneous, 6 years and older
5A15A2F	ECMO	extracorporeal oxygenation, membrane, central, intraoperative
33957	ECMO	extracorporeal membrane oxygenation (ecmo)/extracorporeal life support (ecls) provided by physician; reposition peripheral (arterial and/or venous) cannula(e), percutaneous, birth through 5 years of age (includes fluoroscopic guidance, when performed)

© 2024 Shanklin A et al. *JAMA Network Open.*

33963	ECMO	extracorporeal membrane oxygenation (ecmo)/extracorporeal life support (ecls) provided by physician; reposition of central cannula(e) by sternotomy or thoracotomy, birth through 5 years of age (includes fluoroscopic guidance, when performed)
33981	ECMO	replacement of extracorporeal ventricular assist device, single or biventricular, pump(s), single or each pump
33986	ECMO	extracorporeal membrane oxygenation (ecmo)/extracorporeal life support (ecls) provided by physician; removal of central cannula(e) by sternotomy or thoracotomy, 6 years and older
24376003	ECMO	extracorporeal shockwave lithotripsy of the kidney
33951	ECMO	extracorporeal membrane oxygenation (ecmo)/extracorporeal life support (ecls) provided by physician; insertion of peripheral (arterial and/or venous) cannula(e), percutaneous, birth through 5 years of age (includes fluoroscopic guidance, when performed)
33962	ECMO	extracorporeal membrane oxygenation (ecmo)/extracorporeal life support (ecls) provided by physician; reposition peripheral (arterial and/or venous) cannula(e), open, 6 years and older (includes fluoroscopic guidance, when performed)
33977	ECMO	removal of ventricular assist device; extracorporeal, single ventricle
5A15A2G	ECMO	extracorporeal oxygenation, membrane, peripheral veno-arterial, intraoperative
5A15A2H	ECMO	extracorporeal oxygenation, membrane, peripheral veno-venous, intraoperative

eTable 7: ICD-10 code and diagnosis for each condition that identifies an encounter as eligible for RSV protection during their 2nd RSV season.[1,2]

ICD-10 Code	Diagnosis Description	Diagnosis Class
P27.8	Other chronic respiratory diseases originating in the perinatal period	Chronic respiratory disease in the perinatal period
J96.10	Chronic respiratory failure, unspecified whether with hypoxia or hypercapnia	Chronic respiratory failure
J96.12	Chronic respiratory failure with hypercapnia	Chronic respiratory failure
J96.11	Chronic respiratory failure with hypoxia	Chronic respiratory failure
J96.1	Chronic respiratory failure	Chronic respiratory failure
Q20.0	Common arterial trunk	Congenital heart disease
Q21.0	Ventricular septal defect	Congenital heart disease
Q21.1	Atrial septal defect	Congenital heart disease
Q21.3	Tetralogy of Fallot	Congenital heart disease
Q27.8	Other specified congenital malformations of peripheral vascular system	Congenital heart disease
Q23.1	Congenital insufficiency of aortic valve	Congenital heart disease
Q23.0	Congenital stenosis of aortic valve	Congenital heart disease
Q24.4	Congenital subaortic stenosis	Congenital heart disease
Q25.0	Patent ductus arteriosus	Congenital heart disease
Q21.12	Patent foramen ovale	Congenital heart disease
Q23.4	Hypoplastic left heart syndrome	Congenital heart disease
Q20.4	Double inlet ventricle	Congenital heart disease
Q22.0	Pulmonary valve atresia	Congenital heart disease
Q25.5	Atresia of pulmonary artery	Congenital heart disease
Q24.5	Malformation of coronary vessels	Congenital heart disease
Q25.6	Stenosis of pulmonary artery	Congenital heart disease
Q21.10	Atrial septal defect, unspecified	Congenital heart disease
Q21.19	Other specified atrial septal defect	Congenital heart disease

© 2024 Shanklin A et al. *JAMA Network Open.*

Q25.1	Coarctation of aorta	Congenital heart disease
Q24.9	Congenital malformation of heart, unspecified	Congenital heart disease
Q25.42	Hypoplasia of aorta	Congenital heart disease
Q20.1	Double outlet right ventricle	Congenital heart disease
Q25.21	Interruption of aortic arch	Congenital heart disease
Q24.3	Pulmonary infundibular stenosis	Congenital heart disease
Q27.39	Arteriovenous malformation, other site	Congenital heart disease
Q26.2	Total anomalous pulmonary venous connection	Congenital heart disease
Q20.3	Discordant ventriculoarterial connection	Congenital heart disease
Q25.49	Other congenital malformations of aorta	Congenital heart disease
Q22.4	Congenital tricuspid stenosis	Congenital heart disease
Q24.8	Other specified congenital malformations of heart	Congenital heart disease
Q25.79	Other congenital malformations of pulmonary artery	Congenital heart disease
Q21.2	Atrioventricular septal defect	Congenital heart disease
Q22.5	Ebstein's anomaly	Congenital heart disease
Q20.8	Other congenital malformations of cardiac chambers and connections	Congenital heart disease
Q26.8	Other congenital malformations of great veins	Congenital heart disease
Q26.1	Persistent left superior vena cava	Congenital heart disease
Q20.6	Isomerism of atrial appendages	Congenital heart disease
Q28.2	Arteriovenous malformation of cerebral vessels	Congenital heart disease
Q27.30	Arteriovenous malformation, site unspecified	Congenital heart disease
Q26.3	Partial anomalous pulmonary venous connection	Congenital heart disease
Q27.9	Congenital malformation of peripheral vascular system, unspecified	Congenital heart disease
Q24.0	Dextrocardia	Congenital heart disease
Q25.45	Double aortic arch	Congenital heart disease
Q25.47	Right aortic arch	Congenital heart disease
Q21.11	Secundum atrial septal defect	Congenital heart disease
Q23.3	Congenital mitral insufficiency	Congenital heart disease
Q23.2	Congenital mitral stenosis	Congenital heart disease
Q25.48	Anomalous origin of subclavian artery	Congenital heart disease
Q22.6	Hypoplastic right heart syndrome	Congenital heart disease
Q22.8	Other congenital malformations of tricuspid valve	Congenital heart disease
Q23.8	Other congenital malformations of aortic and mitral valves	Congenital heart disease
Q26.9	Congenital malformation of great vein, unspecified	Congenital heart disease
Q24.1	Levocardia	Congenital heart disease
Q24.6	Congenital heart block	Congenital heart disease
Q22.1	Congenital pulmonary valve stenosis	Congenital heart disease
Q23.9	Congenital malformation of aortic and mitral valves, unspecified	Congenital heart disease
Q28.3	Other malformations of cerebral vessels	Congenital heart disease
Q21.15	Inferior sinus venosus atrial septal defect	Congenital heart disease
Q21.16	Sinus venosus atrial septal defect, unspecified	Congenital heart disease
Q20.2	Double outlet left ventricle	Congenital heart disease
Q20.5	Discordant atrioventricular connection	Congenital heart disease
Q25.4	Other congenital malformations of aorta	Congenital heart disease
Q24.2	Cor triatriatum	Congenital heart disease
Q28.9	Congenital malformation of circulatory system, unspecified	Congenital heart disease
Q21.20	Atrioventricular septal defect, unspecified as to partial or complete	Congenital heart disease
Q25.8	Other congenital malformations of other great arteries	Congenital heart disease

© 2024 Shanklin A et al. *JAMA Network Open.*

Q25.29	Other atresia of aorta	Congenital heart disease
Q25.72	Congenital pulmonary arteriovenous malformation	Congenital heart disease
Q27.1	Congenital renal artery stenosis	Congenital heart disease
Q22.9	Congenital malformation of tricuspid valve, unspecified	Congenital heart disease
Q25.40	Congenital malformation of aorta unspecified	Congenital heart disease
Q22.3	Other congenital malformations of pulmonary valve	Congenital heart disease
Q25.43	Congenital aneurysm of aorta	Congenital heart disease
Q26.4	Anomalous pulmonary venous connection, unspecified	Congenital heart disease
Q21.23	Complete atrioventricular septal defect	Congenital heart disease
Q25.3	Supravalvular aortic stenosis	Congenital heart disease
Q28.8	Other specified congenital malformations of circulatory system	Congenital heart disease
Q25.9	Congenital malformation of great arteries, unspecified	Congenital heart disease
Q26.0	Congenital stenosis of vena cava	Congenital heart disease
Q25.44	Congenital dilation of aorta	Congenital heart disease
Q28.1	Other malformations of precerebral vessels	Congenital heart disease
Q22.2	Congenital pulmonary valve insufficiency	Congenital heart disease
Q21.8	Other congenital malformations of cardiac septa	Congenital heart disease
Q21.13	Coronary sinus atrial septal defect	Congenital heart disease
Q21.22	Transitional atrioventricular septal defect	Congenital heart disease
Q21.9	Congenital malformation of cardiac septum, unspecified	Congenital heart disease
Q21.4	Aortopulmonary septal defect	Congenital heart disease
Q27.0	Congenital absence and hypoplasia of umbilical artery	Congenital heart disease
Q20.9	Congenital malformation of cardiac chambers and connections, unspecified	Congenital heart disease
Q27.32	Arteriovenous malformation of vessel of lower limb	Congenital heart disease
Q26.5	Anomalous portal venous connection	Congenital heart disease
Q25.46	Tortuous aortic arch	Congenital heart disease
Q27.2	Other congenital malformations of renal artery	Congenital heart disease
Q21.14	Superior sinus venosus atrial septal defect	Congenital heart disease
Q24	Other congenital malformations of heart	Congenital heart disease
Q25.2	Atresia of aorta	Congenital heart disease
Q27.33	Arteriovenous malformation of digestive system vessel	Congenital heart disease
Q28.0	Arteriovenous malformation of precerebral vessels	Congenital heart disease
Q25.71	Coarctation of pulmonary artery	Congenital heart disease
Q21.21	Partial atrioventricular septal defect	Congenital heart disease
Q26.6	Portal vein-hepatic artery fistula	Congenital heart disease
E84.0	Cystic fibrosis with pulmonary manifestations	Cystic fibrosis
E84.9	Cystic fibrosis, unspecified	Cystic fibrosis
E84.19	Cystic fibrosis with other intestinal manifestations	Cystic fibrosis
E84.8	Cystic fibrosis with other manifestations	Cystic fibrosis
E84.11	Meconium ileus in cystic fibrosis	Cystic fibrosis
D84.9	Immunodeficiency, unspecified	Immunodeficiency
D82.1	Di George's syndrome	Immunodeficiency
D80.2	Selective deficiency of immunoglobulin A IgA	Immunodeficiency
D82.8	Immunodeficiency associated with other specified major defects	Immunodeficiency
D80.1	Nonfamilial hypogammaglobulinemia	Immunodeficiency
D84.821	Immunodeficiency due to drugs	Immunodeficiency
D89.810	Acute graft-versus-host disease	Immunodeficiency
D81.5	Purine nucleoside phosphorylase PNP deficiency	Immunodeficiency
D89.9	Disorder involving the immune mechanism, unspecified	Immunodeficiency
D89.82	Autoimmune lymphoproliferative syndrome ALPS	Immunodeficiency
D84.89	Other immunodeficiencies	Immunodeficiency

© 2024 Shanklin A et al. *JAMA Network Open.*

D81.810	Biotinidase deficiency	Immunodeficiency
D83.1	Common variable immunodeficiency with predominant immunoregulatory T-cell disorders	Immunodeficiency
D81.1	Severe combined immunodeficiency SCID with low T- and B-cell numbers	Immunodeficiency
D89.89	Other specified disorders involving the immune mechanism, not elsewhere classified	Immunodeficiency
D81.9	Combined immunodeficiency, unspecified	Immunodeficiency
D84.81	Immunodeficiency due to conditions classified elsewhere	Immunodeficiency
D89.813	Graft-versus-host disease, unspecified	Immunodeficiency
D84.8	Other specified immunodeficiencies	Immunodeficiency
D89.811	Chronic graft-versus-host disease	Immunodeficiency
D80.3	Selective deficiency of immunoglobulin G IgG subclasses	Immunodeficiency
D80.8	Other immunodeficiencies with predominantly antibody defects	Immunodeficiency
D89.40	Mast cell activation, unspecified	Immunodeficiency
D84.1	Defects in the complement system	Immunodeficiency
D89.839	Cytokine release syndrome, grade unspecified	Immunodeficiency
D82.4	Hyperimmunoglobulin E IgE syndrome	Immunodeficiency
D81.32	Adenosine deaminase 2 deficiency	Immunodeficiency
D83.9	Common variable immunodeficiency, unspecified	Immunodeficiency
D80.7	Transient hypogammaglobulinemia of infancy	Immunodeficiency
D80.9	Immunodeficiency with predominantly antibody defects, unspecified	Immunodeficiency
D81.39	Other adenosine deaminase deficiency	Immunodeficiency
D81.2	Severe combined immunodeficiency SCID with low or normal B-cell numbers	Immunodeficiency
D89.44	Hereditary alpha tryptasemia	Immunodeficiency
D84.822	Immunodeficiency due to external causes	Immunodeficiency
D89.2	Hypergammaglobulinemia, unspecified	Immunodeficiency
D82.0	Wiskott-Aldrich syndrome	Immunodeficiency
D81.89	Other combined immunodeficiencies	Immunodeficiency
D82.2	Immunodeficiency with short-limbed stature	Immunodeficiency
D80.0	Hereditary hypogammaglobulinemia	Immunodeficiency
D89.832	Cytokine release syndrome, grade 2	Immunodeficiency
D89.831	Cytokine release syndrome, grade 1	Immunodeficiency
D89.834	Cytokine release syndrome, grade 4	Immunodeficiency
D81.7	Major histocompatibility complex class II deficiency	Immunodeficiency
D81.6	Major histocompatibility complex class I deficiency	Immunodeficiency
D80.4	Selective deficiency of immunoglobulin M IgM	Immunodeficiency
D89.812	Acute on chronic graft-versus-host disease	Immunodeficiency
D89.49	Other mast cell activation disorder	Immunodeficiency
D89.42	Idiopathic mast cell activation syndrome	Immunodeficiency
D89.83	Cytokine release syndrome	Immunodeficiency
D80.6	Antibody deficiency with near-normal immunoglobulins or with hyperimmunoglobulinemia	Immunodeficiency
D89.833	Cytokine release syndrome, grade 3	Immunodeficiency
D89.835	Cytokine release syndrome, grade 5	Immunodeficiency
D82.3	Immunodeficiency following hereditary defective response to Epstein-Barr virus	Immunodeficiency
D81.30	Adenosine deaminase deficiency, unspecified	Immunodeficiency
D81.31	Severe combined immunodeficiency due to adenosine deaminase deficiency	Immunodeficiency
D81.818	Other biotin-dependent carboxylase deficiency	Immunodeficiency

© 2024 Shanklin A et al. *JAMA Network Open.*

G71.00	Muscular dystrophy, unspecified	Muscular dystrophy
G71.0	Muscular dystrophy	Muscular dystrophy
G71.01	Duchenne or Becker muscular dystrophy	Muscular dystrophy
G71.09	Other specified muscular dystrophies	Muscular dystrophy
G71.039	Limb girdle muscular dystrophy, unspecified	Muscular dystrophy
M41.40	Neuromuscular scoliosis, site unspecified	Neuromuscular scoliosis
M41.44	Neuromuscular scoliosis, thoracic region	Neuromuscular scoliosis
M41.47	Neuromuscular scoliosis, lumbosacral region	Neuromuscular scoliosis
M41.45	Neuromuscular scoliosis, thoracolumbar region	Neuromuscular scoliosis
M41.46	Neuromuscular scoliosis, lumbar region	Neuromuscular scoliosis
M41.4	Neuromuscular scoliosis	Neuromuscular scoliosis
M41.43	Neuromuscular scoliosis, cervicothoracic region	Neuromuscular scoliosis
P07.15	Other low birth weight newborn, 1250-1499 grams	Prematurity
P07.36	Preterm newborn, gestational age 33 completed weeks	Prematurity
P07.35	Preterm newborn, gestational age 32 completed weeks	Prematurity
P07.39	Preterm newborn, gestational age 36 completed weeks	Prematurity
P07.38	Preterm newborn, gestational age 35 completed weeks	Prematurity
P07.32	Preterm newborn, gestational age 29 completed weeks	Prematurity
P07.30	Preterm newborn, unspecified weeks of gestation	Prematurity
P07.22	Extreme immaturity of newborn, gestational age 23 completed weeks	Prematurity
P07.26	Extreme immaturity of newborn, gestational age 27 completed weeks	Prematurity
P07.24	Extreme immaturity of newborn, gestational age 25 completed weeks	Prematurity
P07.23	Extreme immaturity of newborn, gestational age 24 completed weeks	Prematurity
P07.37	Preterm newborn, gestational age 34 completed weeks	Prematurity
P07.16	Other low birth weight newborn, 1500-1749 grams	Prematurity
P07.14	Other low birth weight newborn, 1000-1249 grams	Prematurity
P07.25	Extreme immaturity of newborn, gestational age 26 completed weeks	Prematurity
P07.17	Other low birth weight newborn, 1750-1999 grams	Prematurity
P07.33	Preterm newborn, gestational age 30 completed weeks	Prematurity
P07.31	Preterm newborn, gestational age 28 completed weeks	Prematurity
P07.20	Extreme immaturity of newborn, unspecified weeks of gestation	Prematurity
P07.21	Extreme immaturity of newborn, gestational age less than 23 completed weeks	Prematurity
P07.34	Preterm newborn, gestational age 31 completed weeks	Prematurity
P07.18	Other low birth weight newborn, 2000-2499 grams	Prematurity
P07.10	Other low birth weight newborn, unspecified weight	Prematurity

© 2024 Shanklin A et al. *JAMA Network Open.*

Supplemental Results

For each encounter, the hospital type, size, and location were documented. Hospital type and size were designated by the database. Hospital location was described using the first digit of its US zip code. Appendix Table 8 shows the number of encounters in each hospital size, type, and location, stratified by the presence or absence of an RSV infection. Appendix Figure 1 (eFigure 1) shows the number of encounters in each physical location.

Encounter demographics and outcomes were also stratified by age. Appendix Table 9 shows the seasonality of all pediatric ICU admissions from January 2017 to December 2019, with encounters categorized as 0-2 years on admission, 2-5 years on admission, and >5 years on admission. Appendix Table 10 shows the demographics and outcomes of all ICU admissions from 2017 to June 2023, stratified in the same age groups. Lastly, Appendix Table 11 shows demographics and outcomes for all ICU encounters with RSV stratified by the receipt of positive pressure ventilation and age. This table used the following age groups: 0-1 years on admission, 1-2 years on admission, 2-5 years on admission, and >5 years on admission.

eTable 8: Hospital Type, Size, and Location of all Pediatric ICU Encounters between January 1st, 2017, and December 31st, 2019, Stratified by RSV Infection

**Demographics** (N, %)	**Total** (119,782)	**RSV** (13,702, 11.4%)	**No RSV** (106,080, 88.6%)
**Hospital Type** N (%)			
Academic	22408 (18.71%)	3292 (24.03%)	19116 (18.02%)
Acute Care or Critical Access	4567 (3.81%)	176 (1.28%)	4391 (4.14%)
Children's	61512 (51.35%)	7118 (51.95%)	54394 (51.28%)
Health System	31295 (26.13%)	3116 (22.74 %)	28179 (26.56%)
**Hospital Size** N (%)			
<200 beds	183 (0.15%)	16 (0.12%)	167 (0.16%)
200-299 beds	49830 (41.60%)	6334 (46.23%)	43496 (41.00%)
300-399 beds	26828 (22.40%)	1496 (10.92%)	25332 (23.88%)
> 500-beds	42941 (35.85%)	5856 (42.74%)	37085 (34.96%)
**1st Digit of Zip Code** N (%)			
0	10843 (9.1%)	1304 (9.5%)	9539 (9.0%)
1	38 (0.0%)	0 (0.0%)	38 (0.0%)
2	19740 (16.5%)	3831 (28.0%)	15909 (15.0%)
3	22154 (18.5%)	2153 (15.7%)	20001 (18.9%)
4	36 (0.0%)	0 (0.0%)	36 (0.0%)
5	222 (0.2%)	4 (0.0%)	218 (0.2%)
6	16995 (14.2%)	503 (3.7%)	16492 (15.6%)
7	16988 (14.2%)	902 (6.6%)	16086 (15.16%)
8	16012 (13.4%)	3525 (25.7%)	12487 (11.77%)
9	16754 (14.0%)	1480 (10.8%)	15274 (14.4%)

© 2024 Shanklin A et al. *JAMA Network Open.*

eTable 9: Seasonality of all Pediatric ICU Encounters between January 1st, 2017, and December 31st, 2019, Stratified by Age

a. Encounters admitted from 2017-2019 were used to represent the RSV season and the RSV peak to minimize alteration by the COVID-19 pandemic.[7]

b. North American RSV season is defined as October-April. The peak is defined as December-January.[8]

Variables	**0-2 years on admission** N (% age group)			**2-5 years on admission** N (% age group)			**2-5 years on admission** N (% age group)		
	**Total**	**RSV**	**No RSV**	**Total**	**RSV**	**No RSV**	**Total**	**RSV**	**No RSV**
**All ICU Encounters[a]**									
Encounters	21620 (100%)	3595 (16.6%)	18025 (83.4%)	9651 (100%)	1024 (10.6%)	8627 (89.4%)	27631 (100%)	1326 (4.8%)	26305 (95.2%)
ICU days, sum (% total)	112889 (100%)	27620 (24.5%)	85269 (75.5%)	36150 (100%)	7107 (19.7%)	29043 (80.3%)	95347 (100%)	13091 (13.7%)	82256 (86.3%)
Hospital days, sum (% total)	183806 (100%)	44172 (24.0%)	139635 (76.0%)	66727 (100%)	12713 (19.1%)	54014 (81.0%)	191337 (100%)	24361 (12.7%)	166977 (87.3%)
**RSV Season[b]**									
Encounters	14507 (100%)	2771 (19.1%)	11736 (80.9%)	5782 (100%)	760 (13.1%)	5022 (86.9%)	15963 (100%)	901 (5.6%)	15062 (94.4%)
ICU Days, sum (% total)	71012 (100%)	18174 (25.6%)	52839 (74.4%)	21627 (100%)	4923 (22.8%)	16704 (77.2%)	57127 (100%)	8528 (14.9%)	48599 (85.1%)
Hospital days, sum (% total)	118397 (100%)	30659 (25.9%)	87738 (74.1%)	39907 (100%)	8573 (21.5%)	31333 (78.5%)	112221 (100%)	15448 (13.8%)	96774 (86.2%)
**RSV Peak[b]**									
Encounters	4777 (100%)	1059 (22.2%)	3718 (77.8%)	1781 (100%)	287 (16.1%)	1494 (83.9%)	4471 (100%)	272 (6.1%)	4199 (93.9%)
ICU days, sum (% total)	23804 (100%)	7174 (30.1%)	16630 (69.9%)	6602 (100%)	1665 (25.2%)	4937 (74.8%)	15143 (100%)	2358 (15.6%)	12786 (84.4%)
Hospital days, sum (% total)	38924 (100%)	11660 (30.0%)	27264 (70.1%)	12318 (100%)	2813 (22.8%)	9505 (77.2%)	31466 (100%)	4428 (14.1%)	27038 (85.9%)

© 2024 Shanklin A et al. *JAMA Network Open.*

eTable 10: Demographics and Outcomes for all Pediatric ICU Encounters between January 1st, 2017, and June 1st, 2023, Stratified by Age

a. RSV and non-RSV encounter characteristics were compared using two-tailed Pearson's x2 tests for categorical variables and two-tailed Wilcoxon rank-sum tests for continuous variables.

b. Primary, public, and unknown insurance types were compared with post-hoc analysis. Public insurance was the reference group.

c. Positive pressure ventilation includes both invasive and non-invasive ventilation.

d. High-risk conditions were identified by ICD codes selected based on CDC's recommendations for nirsevimab administration.[2]

© 2024 Shanklin A et al. *JAMA Network Open.*

	0-2 years on admission N (% age group)				2-5 years on admission N (% age group)				>5 years on admission N (% age group)			
**Variables**	**Total** 42387 (100%)	**RSV** 7212 (17.2%)	**No RSV** 35115 (83%)	***P*-value**[a]	**Total** 20082 (100%)	**RSV** 2557 (12.7%)	**No RSV** 17525 (87.3%)	***P*-value**[a]	**Total** 57313 (100%)	**RSV** 3873 (6.8%)	**No RSV** 53440 (93.2%)	***P*-value**[a]
Male	24730 (58.3%)	4234 (59.5%)	20406 (58.1%)	0.02	11162 (55.6%)	1393 (54.5%)	9769 (55.7%)	0.33	29865 (52.1%)	2067 (53.4%)	27798 (52.0%)	0.26
Age, years median, (IQR)	0.6 (0.3, 1.2)	0.6 (0.2, 1.2)	0.7 (0.3, 1.2)	0.55	3.2 (2.5, 4.0)	3.0 (2.4, 3.9)	3.2 (2.5, 4.0)	<0.001	12.8 (8.8, 15.6)	11.3 (7.6, 14.9)	12.9 (8.9, 15.6)	< 0.001
Weight, kg, median, (IQR)	7.7 (5.2, 10.0)	7.8 (5.4, 10.0)	7.7 (5.2, 10.0)	0.02	14.5 (12.6, 16.8)	14.2 (12.3, 16.5)	14.6 (12.6, 16.9)	<0.001	45.0 (28.1, 62.5)	36.6 (24.7, 57.9)	45.5 (28.5, 62.8)	<0.001
**Primary Insurance**												
Public	26379 (51.0%)	3742 (51.5%)	17869 (50.9%)	REF[b]	10207 (50.8%)	1294 (50.6%)	8913 (50.9%)	REF[b]	26379 (46.0%)	1975 (51.0%)	24404 (45.7%)	REF[b]
Private	14611 (21.7%)	1466 (20.2%)	7740 (22.0%)	0.003[b]	4474 (22.3%)	539 (21.1%)	3935 (22.5%)	0.29[b]	14611 (25.5%)	756 (19.5%)	13855 (25.9%)	<0.001[b]
Other or Unknown	11570 (27.3%)	2064 (28.4%)	9506 (27.1%)	0.23[b]	5401 (26.9%)	724 (28.3%)	4677 (26.7%)	0.20[b]	16323 (28.4%)	1142 (29.5%)	15181 (28.4%)	0.06[b]
**Hospital Type**				0.05				<0.001				<0.001
Children's Hospital	21560 (50.9%)	3624 (49.8%)	17936 (50.1%)		11009 (54.8%)	1315 (51.4%)	9694 (55.3%)		28943 (50.5%)	2179 (56.3)	26764 (50.1%)	
Not a Children's Hospital	20827 (49.1%)	3648 (50.2%)	17179 (48.9%)		9073 (45.2%)	1242 (48.6%)	7831 (44.7%)		28370 (49.5%)	1694 (43.7%)	26676 (49.9%)	
**ICU Length of Stay**												
ICU days per encounter, median (IQR)	2.2 (1.1, 4.7)	2.8 (1.6, 5.8)	2.0 (1.0, 4.4)	<0.001	1.7 (0.9, 3.7)	2.3 (1.3, 5.1)	1.7 (0.9, 3.4)	<0.001	1.6 (0.9, 3.4)	3.3 (1.6, 8.0)	1.6 (0.9, 3.2)	<0.001
Sum of ICU days	226270 (100%)	56491 (25.0%)	169779 (75.0%)		79330 (100%)	17152 (21.6%)	62178 (78.4%)		212178 (100%)	35691 (16.8%)	176488 (83.2%)	
**Hospital Length of Stay**												
Hospital days per encounter, median (IQR)	4.1 (2.3, 8.0)	5.1 (3.0, 10.3)	3.9 (2.2, 7.6)	<0.001	3.3 (1.9, 6.9)	4.6 (2.7, 10.5)	3.2 (1.8, 6.3)	<0.001	3.5 (2.0, 7.1)	7.1 (3.6, 17.2)	3.4 (1.9, 6.7)	<0.001
Sum of hospital days	367431 (100%)	90331 (24.6%)	277100 (75.4%)		141369 (100%)	30921 (21.9%)	110448 (78.1%)		421292 (100%)	68156 (16.2%)	353136 (83.8%)	
**Illness Severity** N (%)												
Respiratory Failure	21392 (50.5%)	5675 (78.0%)	15717 (44.8%)	<0.001	8402 (41.8%)	1871 (73.2%)	6531 (37.3%)	<0.001	14406 (25.1%)	2177 (56.2%)	12229 (22.9%)	<0.001

© 2024 Shanklin A et al. *JAMA Network Open.*

Positive Pressure Ventilation[c]	8996 (21.2%)	2013 (27.7%)	6983 (19.9%)	<0.001	4016 (20.0%)	739 (28.9%)	3277 (18.7%)	<0.001	9883 (17.2%)	1322 (34.1%)	8561 (16.0%)	<0.001
Vasoactive Medication	7158 (16.9%)	1269 (17.5%)	5889 (16.8%)	0.16	3169 (15.8%)	522 (20.4%)	2647 (15.1%)	<0.001	11300 (19.7%)	1266 (32.7%)	10034 (18.8%)	<0.001
ECMO	196 (0.5%)	60 (0.8%)	136 (0.4%)	<0.001	45 (0.2%)	19 (0.7%)	26 (0.2%)	<0.001	146 (0.3%)	45 (1.2%)	101 (0.2%)	<0.001
Death	1712 (4.0%)	251 (3.5%)	1461 (4.2%)	0.005	1066 (5.3%)	149 (5.8%)	917 (5.2%)	0.21	3076 (5.4%)	335 (8.7%)	2741 (5.1%)	<0.001
**High-Risk Condition[d]**	10888 (25.7%)	1371 (18.8%)	9517 (27.1%)	<0.001	3779 (18.8%)	428 (16.7%)	3351 (19.1%)	0.004	8195 (14.3%)	762 (19.7%)	7433 (13.9%)	<0.001
**High-Risk Condition[d]**												
Chronic respiratory failure	1055 (2.5%)	129 (1.8%)	926 (2.6%)	<0.001	726 (3.6%)	77 (3.0%)	649 (3.7%)	0.08	1655 (2.9%)	182 (4.7%)	1473 (2.8%)	<0.001
Perinatal respiratory disease	77 (0.2%)	9 (0.1%)	68 (0.2%)	0.20	38 (0.2%)	1 (0.0%)	37 (0.2%)	0.03	25 (0.0%)	0 (0.0%)	25 (0.1%)	0.06
Congenital heart disease	8209 (19.4%)	915 (12.6%)	7294 (20.8%)	<0.001	2326 (11.6%)	215 (8.4%)	2111 (12.1%)	<0.001	3643 (6.4%)	206 (5.3%)	3437 (6.4%)	0.006
Prematurity	2548 (6.0%)	391 (5.4%)	2157 (6.1%)	0.01	514 (2.6%)	68 (2.7%)	446 (2.5%)	0.73	356 (0.6%)	34 (0.9%)	322 (0.6%)	0.04
Immunodeficiency	597 (1.4%)	150 (2.1%)	447 (1.3%)	<0.001	534 (2.7%)	110 (4.3%)	424 (2.4%)	<0.001	1617 (2.8%)	301 (7.8%)	1316 (2.5%)	<0.001
Muscular Dystrophy	23 (0.1%)	8 (0.1%)	15 (0.0%)	0.025	28 (0.1%)	5 (0.2%)	23 (0.1%)	0.42	240 (0.4%)	15 (0.4%)	225 (0.4%)	0.75
Cystic Fibrosis	61 (0.1%)	10 (0.1%)	51 (0.2%)	0.87	27 (0.1%)	6 (0.2%)	21 (0.1%)	0.14	168 (0.3%)	13 (0.3%)	155 (0.3%)	0.61
Neuromuscular scoliosis	13 (0.0%)	4 (0.1%)	9 (0.0%)	0.19	56 (0.3%)	3 (0.1%)	53 (0.3%)	0.10	1453 (2.5%)	106 (2.7%)	1347 (2.5%)	0.41

© 2024 Shanklin A et al. *JAMA Network Open.*

eTable 11: Demographics and Outcomes for all Pediatric ICU Encounters with RSV between January 1st, 2017, and June 1st, 2023, Stratified by Positive Pressure Ventilation and Age

a. Patients under 1 year on admission and patients under two years on admission with a high-risk condition were considered eligible for RSV prevention.[2]
b. High-risk conditions were identified by ICD codes selected based on CDC's recommendations for nirsevimab administration.[2]

© 2024 Shanklin A et al. *JAMA Network Open.*

	Variables	Total	PPV	No PPV
		**RSV Infection** N (% age group)		
		13702 (100%)	4074 (29.7%)	9628 (70.3%)
**0-1 Years on Admission[a]**	All encounters	4865 (100%)	1358 (27.9%)	3507 (72.1%)
	Age, years median, (IQR)	0.4 (0.2, 0.6)	0.3 (0.1, 0.6)	0.4 (0.2, 0.7)
	Weight, kg, median, (IQR)	6.2 (4.6, 8.0)	5.7 (4.2, 7.8)	6.4 (4.8, 8.2)
	Public Insurance	2515 (51.7%)	799 (58.8%)	1716 (48.9%)
	High-Risk Condition	1019 (20.9%)	446 (32.8%)	573 (16.3%)
	ICU days per encounter, median (IQR)	3.1 (1.8, 6.5)	6.7 (3.0, 14.8)	2.6 (1.6, 4.5)
	Hospital days per encounter, median (IQR)	5.8 (3.5, 11.4)	10.7 (5.2, 23.6)	4.9 (3.1, 8.3)
	Death	145 (3.0%)	85 (6.3%)	60 (1.7%)
**1-2 Years on Admission[a]**	All encounters	2407 (100%)	655 (27.2%)	1752 (72.8%)
	Age, years median, (IQR)	1.4 (1.2, 1.7)	1.4 (1.2, 1.7)	1.4 (1.2, 1.7)
	Weight, kg, median, (IQR)	10.5 (9.4, 11.8)	10.5 (9.2, 11.8)	10.5 (9.4, 11.8)
	Public Insurance	1227 (51.0%)	346 (52.8%)	881 (50.3%)
	High-Risk Condition[b]	352 (14.6%)	143 (21.8%)	209 (11.9%)
	ICU days per encounter, median (IQR)	2.2 (1.4, 4.4)	3.5 (1.8, 11.8)	2.0 (1.3, 3.6)
	Hospital days per encounter, median (IQR)	4.0 (2.6, 8.2)	5.9 (2.9, 19.6)	3.8 (2.4, 6.7)
	Death	106 (4.4%)	60 (9.2%)	46 (2.6%)
**2-5 Years on Admission**	All encounters	2557 (100%)	739 (28.9%)	1818 (71.1%)
	Age, years median, (IQR)	3.1 (2.4, 3.9)	3.1 (2.5, 3.9)	3.0 (2.4, 3.9)
	Weight, kg, median, (IQR)	14.2 (12.3, 16.5)	14.4 (12.3, 16.8)	14.2 (12.3, 16.4)
	Public Insurance	1294 (50.6%)	429 (58.1%)	865 (47.6%)
	High-Risk Condition	428 (16.7%)	183 (24.8%)	245 (13.5%)
	ICU days per encounter, median (IQR)	2.3 (1.3, 5.1)	3.9 (1.7, 11.8)	2.0 (1.2, 3.9)
	Hospital days per encounter, median (IQR)	4.6 (2.7, 10.5)	6.8 (3.1, 19.1)	4.1 (2.6, 8.2)
	Death	149 (5.8%)	77 (10.4%)	72 (4.0%)
**>5 years on admission**	All encounters	3873 (100%)	1322 (34.1%)	2551 (65.9%)
	Age, years median, (IQR)	11.3 (7.6, 14.9)	11.5 (7.8, 14.8)	11.3 (7.6, 15.0)
	Weight, kg, median, (IQR)	36.6 (24.7, 57.9)	35.0 (24.5, 55.0)	37.5 (24.8, 58.9)
	Public Insurance	1975 (51.0%)	756 (57.2%)	1219 (47.8%)
	High-Risk Condition	762 (19.7%)	394 (29.8%)	368 (14.4%)
	ICU days per encounter, median (IQR)	3.3 (1.6, 8.0)	7.1 (2.8, 18.0)	2.5 (1.3, 4.9)
	Hospital days per encounter, median (IQR)	7.1 (3.6, 17.2)	12.7 (5.6, 31.9)	5.5 (3.0, 11.6)
	Death	335 (8.6%)	201 (15.2%)	134 (5.3%)

© 2024 Shanklin A et al. *JAMA Network Open.*

eFigure: Number of Encounters in Each United States Zip Code Region

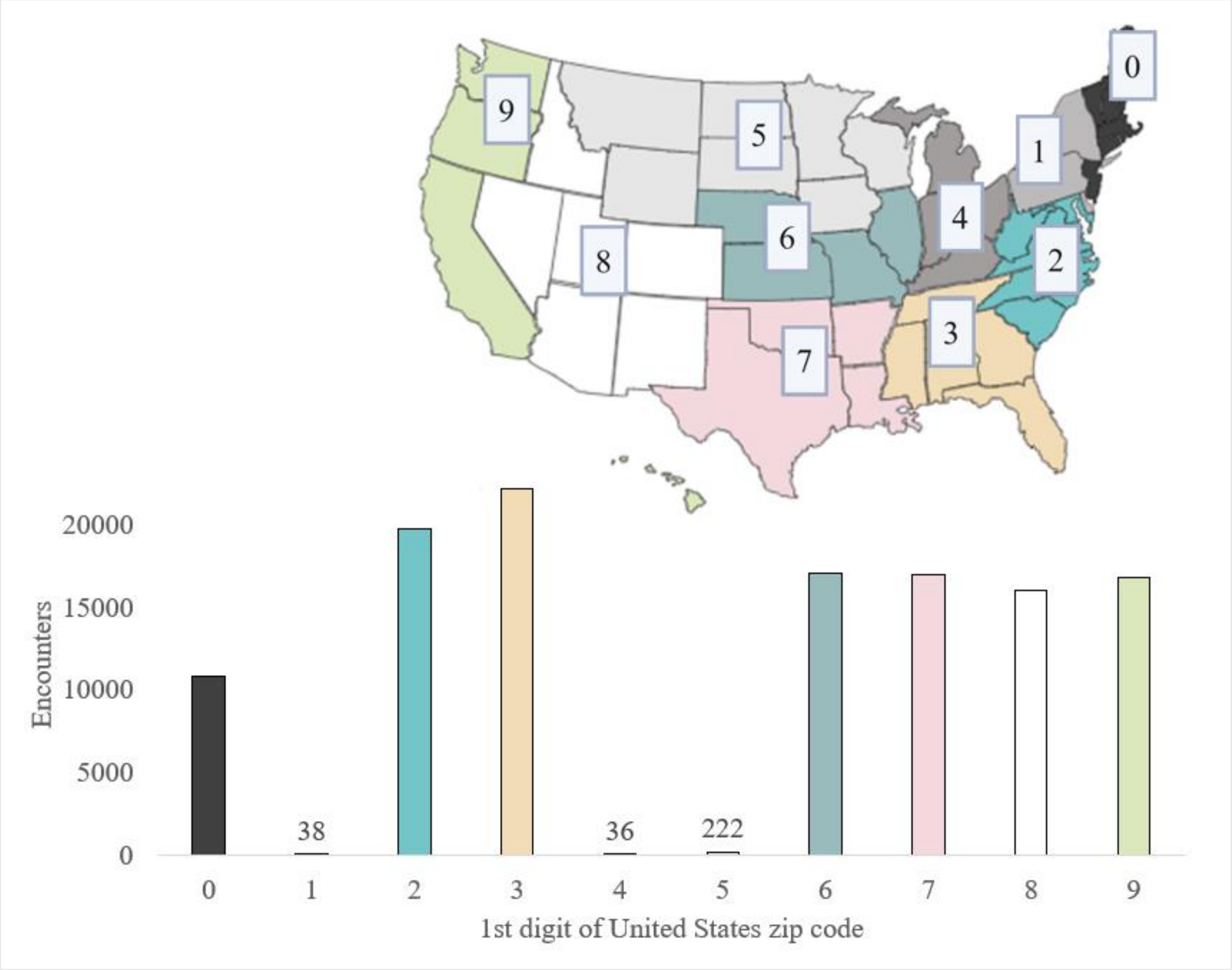

© 2024 Shanklin A et al. *JAMA Network Open.*

eReferences:

1. Obando-Pacheco P, Justicia-Grande AJ, Rivero-Calle I, et al. Respiratory Syncytial Virus Seasonality: A Global Overview. *J Infect Dis*. 2018;217(9):1356-1364. doi:10.1093/infdis/jiy056

2. CDC Health Alert Network. Limited Availability of Nirsevimab in the United States—Interim CDC Recommendations to Protect Infants from Respiratory Syncytial Virus (RSV) during the 2023–2024 Respiratory Virus Season. emergency.cdc.gov.

3. Drysdale SB, Cathie K, Flamein F, et al. Nirsevimab for Prevention of Hospitalizations Due to RSV in Infants. *New England Journal of Medicine*. 2023;389(26):2425-2435. doi:10.1056/NEJMoa2309189

4. Chanapa Tantibanchachai. FDA Approves New Drug to Prevent RSV in Babies and Toddlers. FDA.gov/news.

5. Kampmann B, Madhi SA, Munjal I, et al. Bivalent Prefusion F Vaccine in Pregnancy to Prevent RSV Illness in Infants. *New England Journal of Medicine*. 2023;388(16):1451-1464. doi:10.1056/NEJMoa2216480

6. Hammitt LL, Dagan R, Yuan Y, et al. Nirsevimab for Prevention of RSV in Healthy Late-Preterm and Term Infants. *New England Journal of Medicine*. 2022;386(9):837-846. doi:10.1056/NEJMoa2110275

7. Pelletier JH, Rakkar J, Au AK, Fuhrman D, Clark RSB, Horvat CM. Trends in US Pediatric Hospital Admissions in 2020 Compared With the Decade Before the COVID-19 Pandemic. *JAMA Netw Open*. 2021;4(2):e2037227. doi:10.1001/jamanetworkopen.2020.37227

8. Wang L, Berger N, Davis PB, Kaelber DC, Volkow N, Xu R. Time trend and seasonality in medically attended respiratory syncytial virus (RSV) infections in US children aged 0–5 years, January 2010–January 2023. *Fam Med Community Health*. 2023;11(4):e002453. doi:10.1136/fmch-2023-002453

© 2024 Shanklin A et al. *JAMA Network Open.*
