# Supplementary material for: Respiratory Syncytial Virus and US Pediatric Intensive Care Utilization
Source: JAMA Netw Open. 2024 Oct 25;7(10):e2440997. doi: 10.1001/jamanetworkopen.2024.40997 (PMC11581606; doi:10.1001/jamanetworkopen.2024.40997)
Supplement: Supplement 2. — Data Sharing Statement [file jamanetwopen-e2440997-s002.pdf]

Data Sharing Statement

Shanklin. Respiratory Syncytial Virus and United States Pediatric Intensive Care Utilization. *JAMA Netw Open*. Published October 25, 2024. doi:10.1001/jamanetworkopen.2024.40997

Data
Data available: No

Additional Information
Explanation for why data not available: Data is owned by Oracle-Cerner Real World Data and cannot be made available.
